# The impact of vision impairment on living with congenital aniridia: a pan-European survey study

**DOI:** 10.1186/s13023-026-04419-x

**Published:** 2026-06-02

**Authors:** Renáta Schoffer, Christina Grupcheva, Ivana Kildsgaard, Barbara Poli, Barbara Käsmann-Kellner, Nóra Szentmáry, Erika Maka, Mária Csidey, Annamária Náray, Fabian N. Fries, Zamira Hoxha, Tanja Stachon, Francisco Figueiredo, Jesper Hjortdal, Vito Romano, Claus Cursiefen, Galina Genning, Berthold Seitz, Alejandra Daruich, Dominique Bremond-Gignac, Rosa Sanchez de Vega, Neil Lagali

**Affiliations:** 1 Clinical Department of Ophthalmology, Region Östergötland, Linköping, Sweden; 2 Department of Ophthalmology and Visual Sciences, Medical University, Varna, Bulgaria; 3 Aniridia Europe, Sandefjord, 3214 Norway; 4 Aniridi Sverige, Stockholm, Sweden; 5 Aniridia Italiana, 00149 Rome, Italy; 6 Department of Ophthalmology, Saarland University Medical Center, Homburg/Saar, Germany; 7 Dr. Rolf M. Schwiete Center for Limbal Stem Cell and Congenital Aniridia Research, Saarland University, Homburg/Saar, Germany; 8 Department of Ophthalmology, Semmelweis University, Budapest, Hungary; 9 Department of Ophthalmology, Royal Victoria Infirmary, Newcastle Upon Tyne Hospitals NHS Foundation Trust, Newcastle Upon Tyne, UK; 10 Faculty of Medical Sciences, Bioscience Institute, Newcastle University, Newcastle Upon Tyne, UK; 11 Department of Ophthalmology, Aarhus University Hospital, Aarhus N, Denmark; 12 Eye Unit, Department of Medical and Surgical Specialties, Radiological Sciences, and Public Health, University of Brescia, Viale Europa 15, 25123 Brescia, Italy; 13 Department of Ophthalmology, University of Cologne, Kerpener Str. 62, 50937 Köln, Germany; 14 Asociación Española de Aniridia A.E.A, Madrid, 28040 Spain; 15 INSERM UMRS 1138, Centre de Recherche des Cordeliers, Paris, 75270 France; 16 Ophthalmology Department, Hôpital Universitaire Necker – Enfants Malades, Paris, 75015 France; 17 Department of Biomedical and Clinical Sciences, Linköping University, Linköping, 581 83 Sweden; 18 Department of Ophthalmology, Sørlandet Hospital Arendal, Arendal, Norway; 19 Specialized Eye Hospital, Varna, 9002 Bulgaria

**Keywords:** Congenital aniridia, Quality of life, Survey study, Vision

## Abstract

**Background:**

Congenital aniridia is a rare but severe eye disease stemming from genetic variants in *PAX6* or related genes and affecting all eye structures, causing lifelong disability. Awareness of the disease is poor even among ophthalmology professionals, and how the ocular pathology impacts quality of life for patients is largely unknown. To improve healthcare for this group, a patient perspective is needed. It was therefore the aim of this study to survey subjects with congenital aniridia from across Europe to determine the impact of vision impairment on their daily lives.

**Methods:**

A purpose-developed survey instrument was created and validated by multiple stakeholders and subsequently distributed to individuals and families with congenital aniridia across 15 European countries through European and national aniridia patient associations and the treating ophthalmologists. Survey responses were collected using an online application available in 13 languages or from printed copies. Responses were translated into English and compiled for descriptive and quantitative statistical comparisons.

**Results:**

Of 295 survey respondents (age range 0–79, 58.3% female), photophobia was the most prevalent symptom experienced daily by 83%, followed by 42% experiencing daily dryness, 34% reporting unstable vision daily and 20% experiencing daily ocular pain. 51% of those aged 19 years or younger experienced ocular pain once or more per week, increasing to 61% in adults aged 20 years or older. 93% used a smartphone, of which 55% did not require specialized assistive technology for its use. Most respondents had difficulty recognizing faces, could not read facial expressions, experienced difficulties at school or in the workplace and in social settings, reporting bullying, misunderstandings, social exclusion and isolation. 59% required help at home and 64% outside the home to accomplish daily living tasks, with children requiring help more frequently than adults.

**Conclusions:**

Congenital aniridia is characterized by multiple ocular symptoms experienced frequently, affecting daily living in all age groups. Areas to be addressed include provision of adequate symptom relief and improving inclusion, independence, and quality of life for this rare patient group, particularly for children.

**Supplementary Information:**

The online version contains supplementary material available at 10.1186/s13023-026-04419-x.

## Background

Congenital aniridia is a rare genetic disorder arising principally from variants in the *PAX6* gene that orchestrates eye development [1, 2]. The disease is characterized by abnormal development of diverse ocular and periocular tissues including the Meibomian glands, tear film, cornea, iris, trabecular meshwork, lens, retina and optic nerve [1]. Affected individuals can present with a range of symptoms including reduced visual acuity, pain, photophobia, and failure of the ocular surface (corneal limbal stem cell failure). In most cases, congenital aniridia leads to a progressive worsening of vision with age, although some genotype-phenotype variability exists [3, 4].

Congenital aniridia arises sporadically in about one-third of cases, with the remainder having an autosomal dominant inheritance pattern [5]. Perturbed development of the eye leads to ocular pathologies such as nystagmus, photophobia, ptosis, dry eye disease, keratopathy, iris hypoplasia, glaucoma, cataract, and foveal hypoplasia. Besides ocular effects, aniridia can present as part of a syndrome such as WAGR with more severe ocular symptoms [6, 7], often occurring together with accompanying pathologies in other organs such as the kidneys, pancreas, and brain [1, 8, 9].

Although the structural and clinical spectrum of congenital aniridia is reported widely in the literature [1–3, 10], the effect of aniridia on visual function for daily activities and its impact on quality of life is not known. Although some medical interventions to improve vision are available and helpful (such as use of scleral lenses, autologous serum or tear lubricant eye drops, and surgical procedures to reconstruct or restore the ocular surface), often these do not provide a sustained improvement and are burdensome for patients. There is currently no treatment addressing the genetic or molecular basis of the ocular pathology.

Quality of life indicators of vision, often overlooked in ophthalmology, can provide a useful basis for assessing impacts of a disease and interventions [11, 12]. Prior self-reported data from persons with aniridia are limited to ocular and systemic symptoms in a medical context [13, 14]. Being a rare disease with an estimated prevalence of approximately 1:70,000 [1, 15], a sufficiently large sample is required to provide an accurate representation of the condition, to serve as a basis for assessing and potentially improving the quality of life for patients. We therefore conducted a qualitative pan-European survey to assess vision impairment and its consequences regarding the lived experience of those with congenital aniridia.

## Methods

### Aims, design and setting

The aim of this study was to survey the lived experience of a large and broad geographic sample of persons with congenital aniridia across Europe, to better understand the impact of their vision-related symptoms on their quality of life. The study design was a prospective, qualitative survey study with a purpose-developed survey instrument. The study was initiated as part of the European Union’s Cooperation on Science and Technology (COST) program, under the specific Action CA-18,116, ANIRIDIA-NET (www.aniridia-net.eu). A pan-European Working Group of clinicians, scientists, clinician-scientists, national and European aniridia patient organization representatives collaboratively developed the survey. The study followed the Standards for Reporting Qualitative Research (SRQR) Recommendations [16]. The surveys and study were approved by the Research Ethics Committee of the Medical University of Varna, Bulgaria (Ethical permit no. N 99/14.01.2022г). All subjects gave written informed consent prior to participation, and the study followed the tenets of the Declaration of Helsinki.

The survey (Appendix I) consisted of 21 questions including informed consent, demographic data, symptom frequency, use of aids/devices, functional vision in daily situations, and need for assistance. Free text comments were possible for each question. The survey was developed with input from patient associations, clinicians and researchers within the Working Group. A draft was validated in a group of 30 congenital aniridia subjects in the United Kingdom who provided feedback and suggestions for improvement. The survey was thereafter revised and finalized by the Working Group.

The survey was translated from English into 12 languages (Bulgarian, Danish, Estonian, French, German, Greek, Hungarian, Italian, Norwegian, Russian, Spanish and Swedish). Surveys were made available on an online platform based in Bulgaria, accessible with a digital link to text and text-to-voice versions. Hardcopy was made available for those unable to access or complete the survey online.

Inclusion criteria were congenital aniridia subjects of all ages reachable via national patient associations or treating physicians and willing to complete the survey (parents in the case of young children). The Aniridia Europe association contacted national aniridia associations within Europe to provide information and access to the survey, while physicians directly contacted their patients. Surveys were distributed and collected from September 2022 – September 2023. Age category, sex and country only were requested in the survey to maintain anonymity.

### Data analysis and statistics

Comparison of sex across age groups was performed using the Pearson’s Chi-Square test. Frequencies of pain and photophobia across geographic regions were compared using a binary logistic regression model with the symptom as the dependent variable and frequencies as the weighting factor. IBM SPSS Statistics (Version 29.0.2.0, IBM Corp.) was used for all statistical tests and a P-value of < 0.05 was considered significant.

## Results

### Demographics

**Fig. 1:**
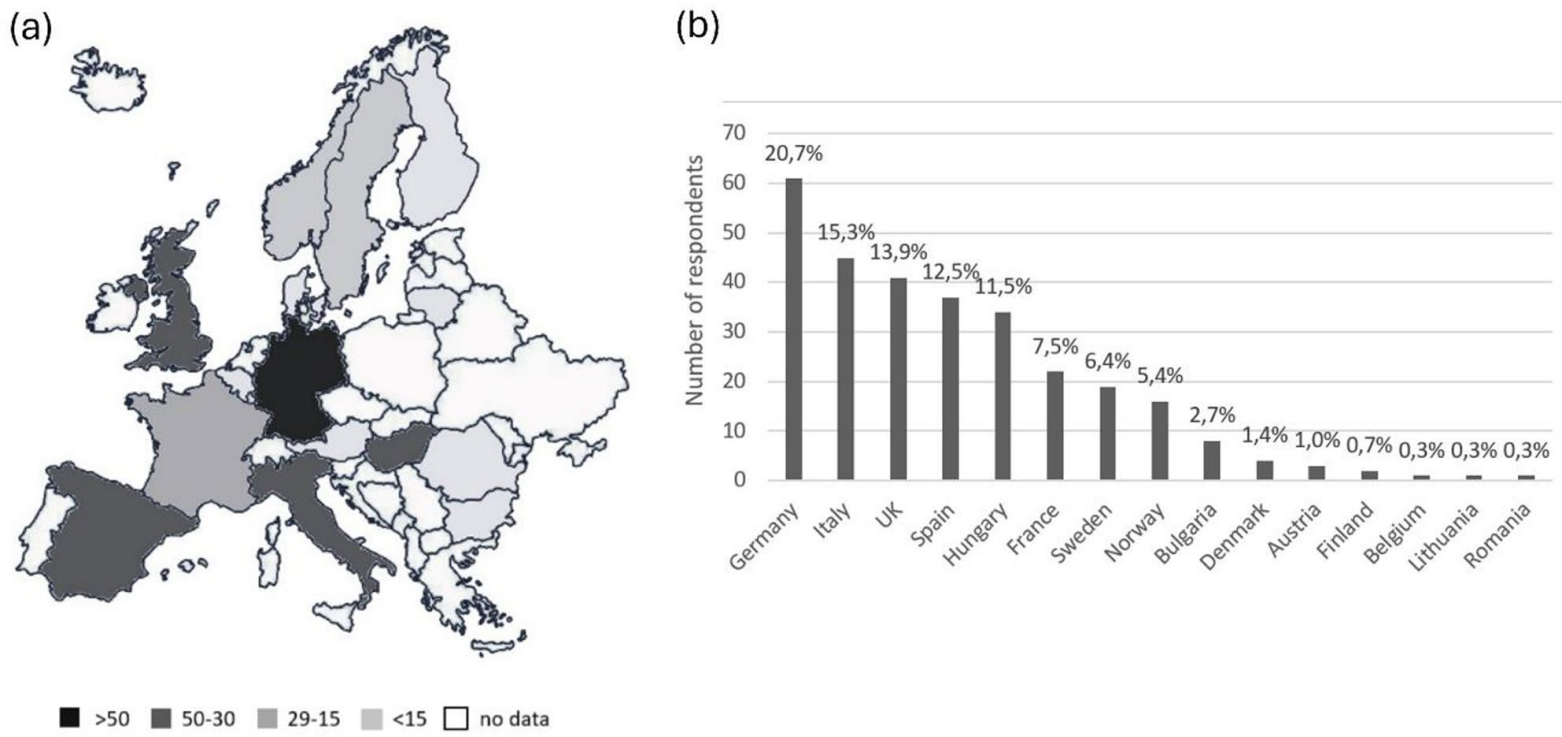
Geographic distribution of survey respondents. (**a**) European map depicting country of residence of respondents and number of respondents coded by grayscale shading. (**b**) Number and percentage of respondents from all participating countries

The survey was completed by persons with congenital aniridia (respondents) in 15 European countries (Fig. 1). Of 368 persons asked to complete the survey by the patient associations or by the treating physicians, 80% (295) completed the survey, and 200/295 (67.8%) were completed digitally. Non-responders were those who were members of a national aniridia association but did not respond to repeated email requests to complete the digital survey. Given an estimated prevalence of 1:70,000 and a combined population in the 15 included countries of 410.9 million (2022; Eurostat), the survey respondents represent an estimated 5% of the aniridia population within Europe.

Age and sex distribution of respondents are given in Table 1. Age ranged from a few weeks to 79 years. Children and young adults in the first two decades of life represented 31.5% of respondents. Of all respondents, 58.3% were female and 41.3% male, with one respondent identifying as non-binary, gender-fluid, or agender. Proportional testing revealed no significant differences in distribution of sex across age categories (*P* = 0.32).

**Table 1:** Distribution of age and sex of respondents of the survey

Age group (years)	Total respondents (%)	Female	Male	Other
0–9	41 (13.9)	21	20	0
10–19	53 (18.0)	33	19	1
20–29	53 (18.0)	28	25	0
30–39	50 (16.9)	28	22	0
40–49	48 (16.3)	30	18	0
50–59	27 (9.2)	16	11	0
60–69	17 (5.8)	14	3	0
70–79	6 (2.0)	2	4	0
80–89	0	0	0	0
Total (%)	295 (100)	172 (58.3)	122 (41.3)	1 (0.003)

### Frequency of symptoms

**Fig. 2:**
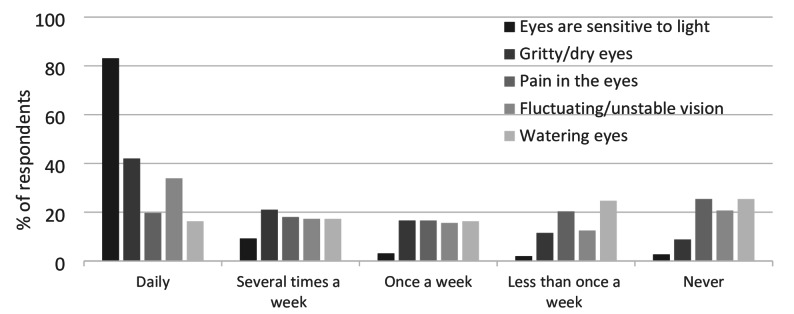
Reported frequency of ocular symptoms across all respondents

Photophobia was the most prevalent symptom (Fig. 2), experienced daily by 83% and at least several times weekly by 92%. 8 respondents (2.7%) reported never experiencing photophobia; however, 4 of these commented they were blind and 2 additional subjects were babies with parents not noting any photophobia from the babies´ reactions. Photophobia was noted by respondents as sometimes being weather-dependent or arising/worsening after ocular surgery. Severity ranged from those who could tolerate indoor lighting to those who were unable to tolerate any direct light or screens without the use of sunglasses. Gritty and/or dry eyes were frequent, with 42% experiencing dryness daily and 63% at least several times weekly. 34% reported unstable or fluctuating vision daily, and 51% at least several times weekly. 75% reported experiencing ocular pain at least occasionally, with 20% experiencing ocular pain daily. 51% of those under the age of 20 and 61% of adults reported ocular pain once or more frequently per week. Ocular pain was noted by some respondents to be related to ocular dryness and ulcers on the ocular surface, while intensity and frequency of ocular pain varied from mild to excruciating to the point where one respondent asked doctors to completely remove their eyes. Watery eyes (epiphora) were less common, with 50% never or seldom experiencing this. Photophobia/dry eye or photophobia/epiphora often occurred together. Binary logistic regression revealed that neither ocular pain (*P* = 0.56) nor photophobia (*P* = 0.06) associated with geographic region within Europe.

### Functional vision and assistive technology

42% of respondents used a normal computer/laptop without any assistive devices, while 51% required either specialized hardware or software. Assistive technology used was built-in Windows magnifier or Apple accessibility functions, dark mode, high-contrast mode, and various text enlargement options. 7% responded that they did not use a computer. Infants and young children using a tablet did not require any devices as reported by parents.

Of all respondents, 93% used a smartphone, with 59% of smartphone users able to use the smartphone without assistive aids and the remaining 41% using assistive software/devices. Most used built-in magnifying functions or apps, text-to-speech and voice assistants. Screen settings were with a dark background and brightness levels were typically dimmed.

50% of all respondents had difficulty understanding others face-to-face and only 27% of all respondents were able to read facial expressions. 45% were sometimes able to read facial expressions, while 28% of respondents could never do so. Distance between speaker and listener was very important for reading expressions but posed a challenge for maintaining personal space. Ambient lighting was also noted as being important. Respondents reported difficulties with understanding irony or sarcasm, not knowing if someone is talking to them if not directly addressed by their name, difficulty in reading hand gestures and body language, difficulty in lip reading, missing if someone winks, misunderstanding a speaker, and difficulty in interpreting silences in a conversation.

Of the respondents, 66% completed education-related questions, and of these 84% reported difficulty completing study-related tasks and 28% noted the difficulties were always present. Older respondents noted many assistive technologies were not available in the past. Difficulties reading handwritten text on paper or blackboard was noted, whereas digital text was easier. Others mentioned difficulties such as reading with speed or following fast speaking, trouble with grasping concepts, and experiencing a cognitive delay. Although the level of education achieved was not a specific question, five adults voluntarily mentioned they were attending or had completed university-level education (minimum of 2.5% of the surveyed adult population). 68% of those studying also experienced difficulties in social situations at school because of their vision, with 16% of these reporting having these difficulties all the time while in school. Respondents expressed difficulties in socializing due to inability to read facial expressions, difficulties in finding friends in the schoolyard and joining in playing activities, experiencing bullying due to problems with eyes/vision, being seen as different before changing to a specialized school, exclusion from activities and difficulties in social situations, especially in larger groups.

63% of respondents had some work or employment experience. Of these, 79% reported difficulties in the workplace with completing work-related tasks because of their level of vision and/or vision-related symptoms. 18% with work experience indicated that the difficulties were always present while 21% indicated never experiencing vision-related difficulties at work. Comments included that ocular pain forced them to retire early, improper lighting in the workplace was an issue, work-related tasks and reading documents takes longer than for colleagues, spreadsheet work is difficult, work requiring driving is impossible, vision aids and devices in the workplace are important, colleagues and managers who are helpful and understanding is important, meetings with a projector are difficult, travel and networking are difficult as it is difficult to recognize people, and in general it is difficult to find a job when one has a visual impairment and/or symptoms such as ocular pain, dryness and photophobia.

66% of respondents with work experience noted difficulties in social situations at work, describing difficulty in recognizing colleagues presenting a social hinder (11 respondents, 6% of those with work experience), leading to misinterpretation as being rude or ignoring others, or invading someone’s personal space to see a face or read a name badge. Four (2% of those working) noted that nonverbal communication between colleagues was impossible to interpret, and as a visually impaired person, one was isolated and had to struggle for years to achieve a good work environment.

### Need for assistance in daily activities

**Fig. 3:**
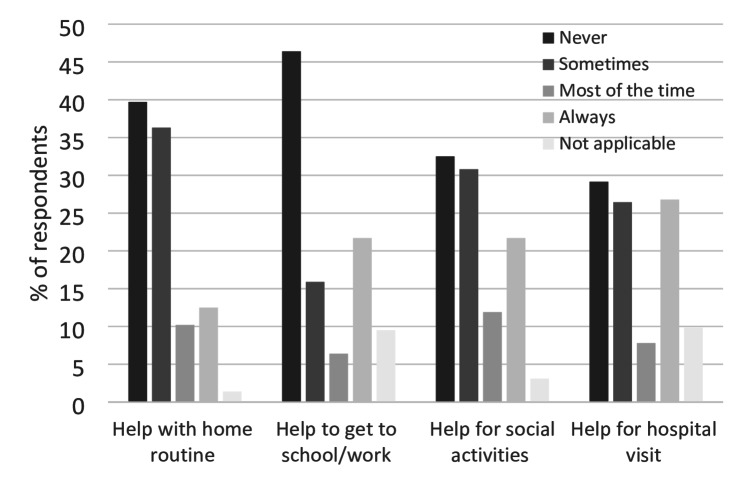
Frequency of those with aniridia needing help from others to perform various activities

40% of all respondents reported never needing help with daily activities while 59% required help (Fig. 3). 46% of respondents, however, did not need help getting to work or school. To participate in social activities such as shopping, sports, restaurants, or events, 33% of all respondents indicated never needing help while 64% required help with for example reading menus or product expiry dates. With WAGR syndrome, help was always needed (e.g. crossing a road), hospital appointments and shopping (reading tags or labels) were particularly difficult, and help was needed even at home. Children with congenital aniridia needed support for vision-related tasks and for cognitive developmental delays associated with learning disabilities. Others reported that cleaning the home is difficult, and lack of depth vision makes the use of stairs difficult. Six (3% of adults) wished to be as independent as possible, however some public transport was difficult or impossible to navigate alone. For all activities, children indicated they always needed help more often than adults over the age of 20.

61% of respondents required help at least some of the time for visiting a doctor, while 27% of respondents always needed help, with bright or dark times of day, familiarity with the route and weather conditions influencing the need for help. Without assistance, physical obstacles or icy conditions in some cases led to falls and injuries. With new or unfamiliar situations, 52% of all respondents needed help always or most of the time, 29% sometimes needed help and 19% never needed help. New situations were disconcerting, particularly outside of daylight hours, requiring help and planning.

**Fig. 4:**
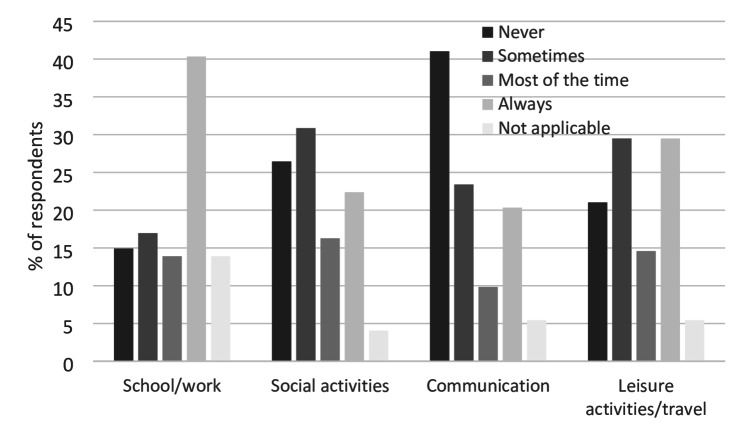
Reported reliance on low vision aids or devices for performing various activities

For school or work, 40% of respondents always relied on assistive low vision aids or devices (non-digital) and only 15% never needed these (Fig. 4). 26% never needed devices in social situations, whereas 70% relied on these at least some of the time. 41% indicated they never required assistive devices beyond their normal mobile telephone for communication, while for leisure and travel 74% used assistive devices at least some of the time. In terms of type of assistive device, some used a blind stick/cane (4 respondents, 1.4%), binoculars or a monocular with their mobile device (8, 2.7%), eyeglasses with filters (3, 1%), sunglasses (4, 1.4%) and various types of magnifiers (38, 13%). One respondent commented that some aids (blind stick, magnifier) are uncomfortable to use in public because of the social stigma associated with them.

Many could distinguish food on the plate (43%), select drinks or food from the kitchen (43%), select clothes (48%), find belongings (34%) and maintain daily personal hygiene (38%) without help or vision aids. Nevertheless, 26, 30, 27, 24, and 23%, respectively used vision aids for these tasks and 6, 5, 7, 8 and 12%, respectively needed help from others. Respondents noted that tasks in a familiar environment (home) were easiest, using strategies like memorizing locations of objects or not requiring to see food, in cases of total blindness.

Regarding the final open-ended question, respondents indicated problems in organs and systems outside the eyes (both WAGR and non-WAGR) as well as providing a unique perspective. Selected comments are given in Table 2.

**Table 2:** Selected comments from respondents

‘Aniridia is a severe social and communication disability, with many physical and psychological daily challenges, which makes life with Aniridia a lifelong daily challenge.’
‘People just don’t get the light sensitivity thing. It’s not just brightness where others see a round light, I see tic-tac-toe shaped light, then you have the auras and halos… a light that might look a centimetre in size to someone else… with all my in built effects it could be 10 or 29 times bigger.’
‘People with aniridia were born with visual impairment and have learnt to do daily tasks in the same way as any baby or child. Our techniques may be different, but end in the correct result. Often we rely on touch or sound cues where average sighted people would use sight. Being creative in your approach to daily living challenges can be exhausting. But is still preferable to being dependent on other people or things.’
‘One of the biggest problems I have as a visually impaired person is changes to my daily routine, for example, if I walk to the bus stop and suddenly there is a change to my route, for example, roadworks, or garbage collection day makes it extremely more difficult to navigate my route. Also, if I go to a friend’s house who decides to move the furniture, I am not used to the new layout of the house. This also causes difficulty. Every day can present new challenges, it is very hard to explain.’
‘I feel people need to be educated on this eye condition, even the blind society hasn’t heard of it.’
‘Not everyone with aniridia will feel and experience the same difficulties. Moreover, the evolution of the disease means that one does not always have the same symptoms throughout one’s life.’
‘I wish for people to see as I do, just for one month, so that they can understand me.’
‘I am a 2nd-3rd generation with aniridia. We are currently experiencing intense social problems, my son who is almost 22 years old sits at home with no training.’
‘We have experienced genetic discrimination when trying to obtain accident insurance. We could not receive this due to aniridia.’
‘I can do almost everything by myself.’
‘I care for myself with help for reading, using toiletries, and for food. I work with an adaptive computer. I travel to well-known destinations fine on my own, but I prefer to be accompanied for the first time to a new location. I have taken part in courses in my local eye department to help train me, and I give talks at my work to promote awareness in the workplace.’

## Discussion

This pan-European survey of the subjective symptoms and daily tasks of living related to vision impairment in congenital aniridia is the largest survey completed by aniridia subjects to date with 295 respondents from 15 European countries. This represents an estimated 5% of all persons in Europe with aniridia; however, the true prevalence of aniridia is unknown and participating associaitons do not have complete records of all aniridia patients. Aniridia can be unrecognized or undiagnosed, in particular in the elderly and the totally blind.

The partial or total iris absence and keratopathy in aniridia [1, 3] likely led to photophobia as the most reported symptom, followed by dry eyes and fluctuating vision. Ocular pain was prevalent across both children and adults, sometimes being severe. Photophobia, dryness and ulcerations, as well as intraocular surgery were noted in conjunction with ocular pain. In an earlier Norwegian study involving 71 aniridia patients [17], prevalence of ocular pain was 62%. In another study, self-reported dry eye was 56% in 99 subjects with aniridia [14]. A Nigerian study with 16 aniridia patients reported poor vision as the most common presenting symptom (78.6%), with photophobia reported in only 14.3% [18]. Although photophobia, fluctuating vision, and ocular pain are consistently acknowledged as symptoms of congenital aniridia [19], there is a lack of prevalence data. This study provides prevalence estimates of symptoms of congenital aniridia in Europe in the largest group to date, and is to our knowledge the only study documenting the temporal frequency of symptoms.

The ability to use digital devices in modern society and their affordability is essential for inclusion in a societal and social context [20], and it appears that those with aniridia are well-connected to digital information. Still, optimal use of technology requires knowledge of accessibility features and the products offering these. Younger participants tend to adopt assistive technology more quickly [21] while older persons often learn at a slower pace, have less access to new technologies, and experience more significant visual impairment as congenital aniridia progresses [22].

As good visual acuity is key to recognizing faces, expressions and for nonverbal communication [23], this is especially difficult in aniridia, affecting the ability to establish relationships, function in social settings and connect with others at a deeper level. This often leads to isolation, which compounds the isolation experienced from the social stigma of low vision or blindness [24]. Here, technological solutions using facial recognition software could be explored, currently an area of active research and development [25]. Isolation at school was a common theme, along with a desire for schools to be better equipped to integrate children with congenital aniridia. The situation was noted to improve for children in specialized schools for the visually impaired, while in adulthood many experienced social challenges and isolation in the workplace. Nonetheless, good integration into the workplace was possible with a supportive employer and workplace environment, active education of colleagues and employers, or only after many years of struggle and adaptation. Many chose occupations favoring one-on-one contact such as massage therapist or physiotherapist.

Adaptation to daily living was high among those with aniridia, but dependent on severity of the visual impairment, with poorer vision necessitating more assistance. Children and young adults not yet having adapted, reported always needing help with tasks of daily life. Even with good adaptation, external factors such as lighting conditions, unfamiliarity with places and situations, and weather conditions made life challenging for all ages. Visual aids and assistive devices, however, were extremely helpful for many individuals in providing a degree of independence. Finding the optimal low vision aid requires individual testing and regular evaluations to assess sufficiency, with a goal of maximizing residual vision while alleviating symptoms, particularly photophobia [26].

Individual reflections from respondents provided a nuanced picture of living with congenital aniridia. An important theme was that congenital aniridia is not isolated to the eye, even in cases of non-syndromic aniridia. Effects in multiple organs such as the pancreas, brain (e.g., autism), and sense of hearing and smell, are starting to emerge in the scientific literature [9, 27–29]. Respondents indicated these effects may equal (or outweigh) poor vision in reducing quality of life. The treating physician authors concur that multiple organ involvement including brain anomalies in congenital aniridia make the disease more difficult to cope with than other types of congenital visual impairment. A limitation of this study is that it is self-assessment without correlation to objective measures such as degree of visual impairment and other clinical or genotypic parameters. Significant intrafamilial variability with variable expressivity of disease features has been reported [30], and the disease is dynamic, progressively worsening with age in most cases [10, 31]. Future studies should include self-reported measures along with clinical data.

Finally, some positive outcomes were noted, such as a university education, entering the workplace and engaging in meaningful and fulfilling work. Open-ended responses provide a unique insight into this rare disease. Further studies for example, using narrative medicine [32, 33], could improve our understanding.

## Conclusions

From a survey of 295 persons with congenital aniridia from across Europe, the main self-reported symptoms experienced daily were photophobia, dry eyes, unstable vision and eye pain, with these symptoms affecting all ages. Areas to be addressed include providing symptom relief, addressing social and emotional needs, and promoting inclusion and independence. The existence of non-ocular pathologies in congenital aniridia impacting quality of life warrants further research.

## Supplementary Information

Below is the link to the electronic supplementary material.

Supplementary Material 1

## Data Availability

The datasets generated and/or analysed during the current study are not publicly available as data sharing of the responses was not specifically stated in the ethics application or consent form.
