# Supplementary material for: The impact of vision impairment on living with congenital aniridia: a pan-European survey study
Source: Orphanet J Rare Dis. 2026 Jun 2;21:265. doi: 10.1186/s13023-026-04419-x (PMC13445906; doi:10.1186/s13023-026-04419-x)
Supplement: Supplementary file 1 [file 13023_2026_4419_MOESM1_ESM.docx]

**Appendix I – Pan-European Aniridia Survey**

Welcome Page

The EU COST Action ANIRIDIA-NET is planning to conduct a survey study to better understand how visual impairment of people living with Aniridia affects their daily quality of life.

The goal is to better inform doctors, researchers and others about the most important issues concerning Aniridia and visual impairment, from the perspective of persons living with Aniridia.

Your feedback is very important, and we thank you for your time in completing this first Aniridia survey in the EU.

Privacy Information

**About the project**

The EU COST Action ANIRIDIA-NET is planning to conduct a survey study to better understand how visual impairment of people living with aniridia affects their daily quality of life. The instrument is translated into multiple languages and persons with aniridia across Europe are invited to participate.

**Who are we?**

The EU COST Action ANIRIDIA-NET (www.aniridia-net.eu) is a not-for-profit network of medical professionals, researchers, and other stakeholders interested in improving the quality of life of persons living with aniridia. The Action is funded by the European Commission’s COST (Cooperation in Science and Technology) Program.

**Why are we contacting you?**

Because you have Aniridia and we would like to hear from you about your daily experiences.

**What personal data do we hold?**

From data provided by the national aniridia associations, we have collated information on potential participants including name and email address so that we can invite you to participate. We are holding this data in compliance with Article 14 of the GDPR.

**Who is handling this information?**

Working Group 6 members from the COST Action ANIRIDIA-NET, led by Professor Christina Grupcheva (data controller), are responsible for personal information in relation to this project. Working Group members are also serving as the data processors. The data we are collecting is on behalf of ANIRIDIA-NET and will be processed in line with the privacy policy of the Medical University - Varna in Varna, Bulgaria.

**What is the legal basis for processing your data?**

The personal data we are processing includes your contact details that have been shared with us by the national aniridia associations. We are using these solely to invite you to participate in this study and to manage data collection. We do not anticipate that your rights and freedoms will be affected as a result of our use of your data in this way. We are therefore processing your data on the basis that it is within the legitimate interests of the COST Network ANIRIDIA-NET to undertake this task.

Any responses to the survey will be confidential and your contact details will be separated from your responses prior to analysis. We will not ask you to enter your contact details as part of the survey process.

**How long will we keep this data?**

Working Group 6 from ANIRIDIA-NET will retain your contact details for up to one year after the end of the research project, after which time your contact details will be deleted from the project files.

**What are your rights?**

You have the right to request access to and rectification or erasure of personal data. You can also request us to place restrictions on our processing of your data and have the right to object to our processing as well as the right to data portability. To exercise any of these rights or if you have any questions, please contact Professor Christina Grupcheva as the data controller.

**How do you contact us to exercise these rights?**

You may contact Professor Grupcheva at cgrupcheva@gmail.com, citing “ANIRIDIA-NET Survey” in the subject line.

**What happens if I agree to participate in the survey?**

We would like to ask you to complete the survey to help us understand the current challenges facing persons living with aniridia, with a specific focus on visual impairment and its effect on daily activities. Responses to the survey will be confidential. We will not ask you to enter your contact details as part of the process.

We hope you will be happy to help us with this important study for the COST Action ANIRIDIA-NET.

If you would like any further information regarding the project, please contact Professor Christina Grupcheva at cgrupcheva@gmail.com.

Information about survey completion

## The survey should take no more than 15 minutes of your time. Your participation is entirely voluntary, and you may choose to change your mind about participating at any time before, during or after the survey. You do not have to give any reasons for withdrawing but we draw your attention to your rights to delete your data at the bottom of this form.

## As outlined in the privacy information notice, the data you provide will be kept until 12 months after the project ends. The data you provide will be deleted at this point and the analyzed data will be kept indefinitely in an anonymized form. It is our legitimate interest to obtain and retain information on your name and email address, as this is necessary to facilitate contact with you for the project and in doing so does not affect your rights or freedoms. Retention of your information is subject to your rights below. All records will be kept in compliance with the General Data Protection Regulation (GDPR) 2018. This survey will be conducted by the COST Action Working Group 6 and data will be recorded and stored in accordance with institutional procedures at the Medical University of Varna, Bulgaria.

## Your legal rights

## This study is conducted in accordance with EU law including GDPR. You are provided with certain rights that you may have the right to exercise through us. In summary those rights are:

## (1) To access, correct or erase your data;

## (2) To object to the processing of your data;

## (3) To request that our processing of your data is restricted;

## (4) To request that your data be transferred;

## (5) To withdraw your consent for us to process your data.

## If you wish to exercise any of these rights, please contact Professor Christina Grupcheva at cgrupcheva@gmail.com.

## Consent form to participate in survey

### **1. I confirm that I have read the information sheet on participation in the survey.**

-Yes
-No

### **2. I understand that my participation is voluntary and that I am free to withdraw at any time without giving any reason, without being affected in any way or my legal rights being affected.**

-Yes
-No

### **3. I agree to participate in this survey.**

-Yes
-No

## Demographic information

### **4. Please select the country in which you live.**

-Austria
-Belgium
-Bulgaria
-Cyprus
-Croatia
-Czech Republic
-Denmark
-Estonia
-Finland
-France
-Germany
-Greece
-Hungary
-Ireland
-Italy
-Latvia
-Lithuania
-Luxembourg
-Malta
-Netherlands
-Poland
-Portugal
-Romania
-Slovakia
-Slovenia
-Spain
-Sweden
-United Kingdom

### **5. What is your age group?**

-under 10
-10 - 19
-20 - 29
-30 - 39
-40 - 49
-50 - 59
-60 – 69
-70 – 79
- 80+

### **6. What is your gender?**

-Female
-Male
-Prefer to self-describe as (non-binary, gender-fluid, agender, or other)

### **7. How often do you experience the following conditions in your eye(s)?**

|  | **Every day** | **Several times in a week** | **Once in a week** | **Less than once a week** | **Never** |
| --- | --- | --- | --- | --- | --- |
| **My eyes are sensitive to light** | x |  |  |  |  |
| **My eyes are gritty/dry** |  |  |  |  |  |
| **I have pain in my eyes** |  |  |  |  |  |
| **My vision is fluctuating/not stable** |  |  |  |  |  |
| **My eyes are watering** |  |  |  |  |  |

Comments regarding these questions (optional):

### **8. Can you use a regular computer, without special software or devices for the computer to aid in viewing the screen and using the computer?**

-Yes
-No, I need special devices or software to use the computer
-Not applicable

Comment (optional): ______________________________________

### **9. Can you use a regular mobile phone, without special software or devices for the phone to aid in viewing the screen and using the mobile phone?**

-Yes
-No, I need special devices or software to use the mobile phone
-Not applicable

Comment (optional): ______________________________________

### **10. Do you have problems understanding others during face-to-face communication (in person or during online meetings)?**

-Always
-Sometimes
-Never

Comment (optional): ______________________________________

### **11. Are you able to read or interpret facial expressions when talking to someone face-to-face?**

-Always
-Sometimes
-Never

Comment (optional): ______________________________________

### **12. Do you experience difficulty at school, with understanding or completing study-related tasks, because of your vision?**

-Always
-Sometimes
-Never
-Not applicable

Comment (optional): ______________________________________

### **13. Do you experience difficulty at school in social situations with classmates or peers, because of your vision?**

-Always
-Sometimes
-Never
-Not applicable

Comment (optional): ______________________________________

### **14. Do you experience difficulty at work, with understanding or completing work-related tasks, because of your vision?**

-Always
-Sometimes
-Never
-Not applicable
- Comment (optional): ______________________________________

### **15. Do you experience difficulty at work in social situations with colleagues, because of your vision?**

-Always
-Sometimes
-Never
-Not applicable

Comment (optional): ______________________________________

**16. How much help or assistance do you need in your daily life? This could be from a friend, relative, or other person.**

|  | **Always** | **Most of the time** | **Sometimes** | **Never** | **Not applicable** |
| --- | --- | --- | --- | --- | --- |
| **I need someone to help me with my daily routine at home** |  |  |  |  |  |
| **I need someone to help me to go to school/work** |  |  |  |  |  |
| **I need someone to help me, so I can participate in social activities (shopping, sports, restaurants, events)** |  |  |  |  |  |

Comments regarding these questions (optional):

### **17. Do you need help or assistance from someone to attend a doctor’s appointment?**

-Always
-Most of the time
-Sometimes
-Never
-Not applicable

Comment (optional): ______________________________________

### **18. Do you need help or assistance from someone, when you need to do something new or out of the ordinary, such as going to a new place or trying a new task?**

-Always
-Most of the time
-Sometimes
-Never
-Not applicable

Comment (optional): ______________________________________

**19. How often do you need or rely on low vision devices/aids for the following activities?**

|  | **Always** | **Most of the time** | **Sometimes** | **Never** | **Not applicable** |
| --- | --- | --- | --- | --- | --- |
| **School/work** |  |  |  |  |  |
| **Social activities** |  |  |  |  |  |
| **Communication including phone calls** |  |  |  |  |  |
| **Leisure activities or travel** |  |  |  |  |  |

Comments regarding these questions (optional):

**20. To what extent does your vision affect the following daily activities?**

|  | **I always need help** | **I sometimes need help** | **I can manage by myself with vision aids** | **I can manage by myself without vision aids** | **Not applicable** |
| --- | --- | --- | --- | --- | --- |
| **Distinguishing food on my plate** |  |  |  |  |  |
| **Selecting my drinks or food from the kitchen** |  |  |  |  |  |
| **Selecting my clothes** |  |  |  |  |  |
| **Finding my belongings (keys, phone, etc.)** |  |  |  |  |  |
| **Maintaining my daily hygiene (makeup, shaving, cutting nails, etc.)** |  |  |  |  |  |

Comments regarding these questions (optional):

**21. Do you have any further comments?**

End Page

Thank you for completing this survey. Your experiences in living with Aniridia are important for us, and your cooperation is much appreciated.
